# Reliability and Diagnostic Performance of CT Imaging Criteria in the Diagnosis of Tuberculous Meningitis

**DOI:** 10.1371/journal.pone.0038982

**Published:** 2012-06-29

**Authors:** Hugo Botha, Christelle Ackerman, Sally Candy, Jonathan A. Carr, Stephanie Griffith-Richards, Kathleen J. Bateman

**Affiliations:** 1 Division of Neurology, Tygerberg Hospital, University of Stellenbosch, Cape Town, Western Cape, South Africa; 2 Department of Radiodiagnostics, Tygerberg Hospital, University of Stellenbosch, Cape Town, Western Cape, South Africa; 3 Department of Radiology, Groote Schuur Hospital, University of Cape Town, Cape Town, Western Cape, South Africa; Aga Khan University, Pakistan

## Abstract

**Introduction:**

Abnormalities on CT imaging may contribute to the diagnosis of tuberculous meningitis (TBM). Recently, an expert consensus case definition (CCD) and set of imaging criteria for diagnosing basal meningeal enhancement (BME) have been proposed. This study aimed to evaluate the sensitivity, specificity and reliability of these in a prospective cohort of adult meningitis patients.

**Methods:**

Initial diagnoses were based on the CCD, classifying patients into: ‘Definite TBM’ (microbiological confirmation), ‘Probable TBM’ (diagnostic score ≥10), ‘Possible TBM’ (diagnostic score 6–9), ‘Not TBM’ (confirmation of an alternative diagnosis) or ‘Uncertain’ (diagnostic score of <6). CT images were evaluated independently on two occasions by four experienced reviewers. Intra-rater and inter-rater agreement were calculated using the kappa statistic. Sensitivities and specificities were calculated using both ‘Definite TBM’ and either ‘Definite TBM’ or ‘Probable TBM’ as gold standards.

**Results:**

CT scan criteria for BME had good intra-rater agreement (κ range 0.35–0.78) and fair to moderate inter-rater agreement (κ range 0.20–0.52). Intra- and inter-rater agreement on the CCD components were good to fair (κ  =  ranges 0.47–0.81 and 0.21–0.63). Using ‘Definite TBM’ as a gold standard, the criteria for BME were very specific (61.5%–100%), but insensitive (5.9%–29.4%). Similarly, the imaging components of the CCD were highly specific (69.2–100%) but lacked sensitivity (0–56.7%). Similar values were found when using ‘Definite TBM’ or ‘Probable TBM’ as a gold standard.

**Discussion:**

The fair to moderate inter-rater agreement and poor sensitivities of the criteria for BME suggest that little reliance should be placed in these features in isolation. While the presence of the CCD criteria of acute infarction or tuberculoma(s) appears useful as rule-in criteria, their absence is of little help in excluding TBM. The CCD and criteria for BME, as well as any new criteria, need to be standardized and validated in prospective cohort studies.

## Introduction

Tuberculous meningitis (TBM) has a case fatality rate of 15–68% [1]–[3], and more than half of the survivors are left with neurological sequelae [4]. Central nervous system involvement is estimated to occur in around 1% of patients with active tuberculosis [5]. In the Western Cape, an area with a high incidence of tuberculosis, TB is one of the most common causes of meningitis [6].

Early diagnosis and commencement of treatment confers a significantly better prognosis [2], [4]. However, the diagnosis of TBM is complicated by its variable clinical presentation and the lack of specificity of its radiological and laboratory features [7]. For this reason, in most cases, treatment is initiated before a microbiological diagnosis is confirmed. Physicians have to rely on the clinical presentation, and the results of investigations that are available within hours, most notably cerebrospinal fluid (CSF) biochemistry and cell counts and chest radiography [8]. Although brain imaging is not mandatory for a diagnosis of probable or definite TBM, characteristic abnormalities on imaging have been reported to contribute to the diagnostic certainty. A recent expert consensus case definition (CCD) for use in research incorporates the following radiological signs in the diagnosis of TBM: hydrocephalus, infarcts, tuberculoma(s), basal meningeal enhancement, and the presence of pre-contrast basal hyperdensities [9]. On computed tomography (CT) of the brain, hydrocephalus and meningeal enhancement are reported to be the most sensitive signs of TBM, being present in 80% and 75% of paediatric cases [9]–[11], and in 45% and up to 34% of adult cases, respectively [4], [10], [11]. Although magnetic resonance imaging is superior to CT imaging in the diagnosis of TBM [1], this modality may not be readily accessible in resource limited settings with high TB burden.

Furthermore, objective criteria for diagnosing basal meningeal enhancement on CT have recently been proposed based on case-control studies of childhood TBM in the Western Cape [12]–[14]. The authors reported a high sensitivity and specificity for basal enhancement (89% and 94%, respectively) as well as reasonable sensitivities/specificities for hydrocephalus and acute infarction (68%/72% and 62%/78%, respectively). They proposed nine criteria that suggested basal meningeal enhancement. In their paediatric case-control study, the presence of one or more of these criteria provided a sensitivity of 91% and a specificity of 97% in microbiologically confirmed cases of TBM.

The development of clearly defined criteria for the diagnosis of TBM is crucial, and both the CCD and abovementioned criteria for basal meningeal enhancement could greatly aid research into TBM. However, they have not been assessed prospectively. The aim of the present study was to evaluate the sensitivity, specificity inter- and intra-rater reliability of the abovementioned diagnostic CT criteria for basal enhancement, and the imaging components proposed in the CCD for the diagnosis of definite or probable TBM in a well characterised, prospective cohort of adult patients presenting with clinical meningitis.

## Methods

Ethical approval was obtained from the Health Research Ethics Committee at Stellenbosch University (N10/05/174), and the project was conducted in accordance with the Declaration of Helsinki. Written informed consent was obtained from patients who were fully conscious. In patients who were unable to provide consent, written, informed consent was obtained from a relative, or, if no relative was available, two independent physicians. All participants, or, if relevant, their consenting relative, were given an original copy of the signed and dated patient information leaflet and consent form. Written consent was obtained from the next of kin, carers or guardians on behalf of minors involved in the study. All of the above forms of consent were approved by the Health Research Ethics Committee at Stellenbosch University (N10/05/174).

### Participants

Participants in this study are a subgroup of patients enrolled in the Adult Meningitis Study (AMS) currently underway at the Tygerberg Hospital, Cape Town, South Africa between June 2010 and May 2011 in whom a CT brain scan with contrast was performed as part of the diagnostic workup. The AMS aims to enrol all patients presenting to the hospital with clinical features of meningitis, with the goal of improving the early diagnosis of TBM by evaluating current methods of diagnosis and developing a diagnostic approach appropriate to the setting of high TB and HIV prevalence. The inclusion criteria for this cohort are: a clinical suspicion of meningitis, age equal or greater than 15 years and an abnormal CSF with a cell count (>5 white cells per high power field) and/or total protein (≥0.6 g/ml). Exclusion criteria include contraindication(s) to lumbar puncture, failure to obtain an adequate volume of CSF (>5 ml), the diagnosis of subarachnoid haemorrhage (SAH), more than 4 doses of anti-tuberculous (anti-TB) therapy in the last 14 days or more than two doses of a third generation cephalosporin in the past 48 hours. These criteria were derived from recent similar cohorts [15]–[18]. Large volumes of CSF and appropriate ancillary investigations are obtained to ensure that a definitive diagnosis is obtained in as many patients as possible [19]. Imaging studies of the brain are performed according to clinical indication by the attending physician. In each case, based on presenting features, an initial admission diagnosis is made. Patients are managed in the hospital according to routine practice by hospital physicians but followed up at the Neuroinfectious Disease clinic (or telephonically) after 3 months when a final diagnosis is made.

### Diagnostic Classification

In each patient the initial diagnosis was based on the CCD, which categorises patients as: ‘Definite TBM’ – positive microbiology (smear, culture or PCR) confirming TBM; ‘Not TBM’ – the confirmed presence of an alternative infectious diagnosis and no suggestion of dual infection; ‘Probable TBM’ and ‘Possible TBM’ – based on the total number of points obtained based on the clinical findings, CSF parameters, the presence of tuberculosis elsewhere and cerebral imaging criteria (see supporting information Table S1 for more details regarding the diagnostic classification). An adjusted scoring system applies in patients in whom cerebral imaging is not available, and this was used to classify patients in order to avoid bias. Patients who were not treated for tuberculosis, and made a full recovery at three months follow up were classified as ‘Not TBM’. Participants who had insufficient criteria for a diagnosis of ‘Possible TBM’ were recorded as ‘Uncertain’.

### Imaging

All but 3 CT scans were performed on a Siemens SOMATOM Emotion 6 scanner. Two of the former were performed on a Toshiba Aquilion, and one on a Siemens SOMATOM Sensation 40 apparatus. The scanning technique and dose of intravenous contrast medium (1 ml/kg body weight) were standardised. Images were reviewed for purposes of classification on diagnostic monitors using Philips Brilliance Workspace Portal v 2.6.0.18.

Each set of CT images were reviewed independently on two separate occasions, two to three weeks apart, by two consultant radiologists, a neuroradiologist and a neurologist - a total of 8 assessments. The reviewers were blinded to the diagnostic category of the subject, as well as to the other reviewers’ reports. The scans were assigned random numbers, and all patient information removed apart from date of birth. During the interval between viewings, new random numbers were assigned to each set of images to remove a recall bias. Reviewers were provided with a standard reporting form with the CCD and criteria for the presence of basal enhancement, and their briefing included the viewing of examples of each of the criteria, and standard instructions on how the reporting form should be completed. They reported the presence or absence of each CCD criterion and, for the category ‘hydrocephalus’ and ‘infarction’, an additional category of ‘questionable’ was provided. The nine criteria for basal enhancement (BE) are [12], [14]:

1. *Contrast filling the cisterns*, with obliteration of CSF that surrounds normal vascular enhancement
2. *‘Double and triple line signs’*–identification of two or three lines of enhancement in the middle cerebral artery cisterns represents enhancement of the meninges lining the lobes that lie against each other (frontal and temporal lobes) with and without visible enhancement of the middle cerebral artery itself, respectively. This sign should not be assessed at the distal middle cerebral artery where it divides into its sylvian branches
3. *‘Linear enhancement’* in the middle cerebral artery cistern seen over two or more contiguous slices. (The middle cerebral artery itself is too small to be seen in its full horizontal length over more than one slice and is usually tortuous and therefore is seen in an interrupted fashion on one slice and not as linearity.)
4. The *‘Y’ sign* at the junction of the suprasellar cistern and middle cerebral artery cistern. Pure vessel enhancement at this region lacks an arm of the ‘Y’ because the posterior cerebral artery is not often seen on CT, as it is small
5. Enhancement of the posterior aspect of the *infundibular recess of the third* ventricle in the suprasellar cistern. There is no known vessel that lies here that can be confused with meningeal enhancement
6. *Ill-defined edge* to the enhancement as opposed to sharply marginated enhancement of normal vessels
7. ‘*Join the dots’*–normal enhancement of the Sylvian vessels is seen as separate dots, as the branches are seen in cross section. Abnormal enhancement is present when the dots are joined by linear enhancement
8. *Nodular enhancement*–is always pathological because normal meninges are smooth
9. *Asymmetry* of any of the above

### Intra-rater reliability

Intra-rater reliability was calculated using the kappa statistic. For the intra-rater reliability of the variables ‘acute infarction’ and ‘hydrocephalus’, weighted kappa statistics were calculated, with the disagreements between a normal result (‘no’) and either of the abnormal results (‘questionable’ and ‘definitely’) more heavily penalised than disagreement between the abnormal results. Furthermore, a variable representing the presence of at least two of the criteria for basal meningeal enhancement was derived (‘All BE’, see below). Confidence intervals (95%) were calculated from the standard error. Given the paradoxical kappa values that can occur as a result of underlying observer bias or asymmetry of the marginal totals, the maximal obtainable value of kappa for the given marginal totals (Kmax) was calculated [20], along with the bias index (BI), prevalence asymmetry index (PAI) and prevalence and bias adjusted kappa (PABAK) [21].

### Inter-rater reliability

Inter-rater reliability was calculated with Fleiss’ kappa statistic. The two ordinal variables were collapsed into binary variables (‘no’ and ‘questionable/definitely’), given that questionable findings would be more likely, in this clinical context, to influence clinical management in a similar way to definite findings. Again, the variable representing the presence of at least two of the criteria for basal meningeal enhancement was included. Bias corrected confidence intervals were derived through bootstrapping, with 10 000 repetitions, as described by Reichenheim [22].

Kappa was interpreted in the following manner: Poor agreement  =  Less than 0.20, fair agreement = 0.20 to 0.40, moderate agreement = 0.40 to 0.60, good agreement = 0.60 to 0.80 and very good agreement = 0.80 to 1.00 [23].

### Diagnostic Performance

A BE feature was taken as present if it was recorded as present in more than 4 of the 8 reviewer’s ratings. The derived variable, ‘All BE’, was considered present if at least 2 of the 9 signs of basal meningeal enhancement were reported in more than 4 of 8 ratings. Sensitivity and specificity for each of the variables were calculated in cases with ‘Definite TBM’ compared to cases diagnosed as ‘Not TBM’. In a separate analysis, sensitivity and specificity were calculated, for cases of either ‘Definite TBM’ or ‘Probable TBM’ compared to cases diagnosed as ‘Not TBM’ or ‘Uncertain’. The proportion of positive findings that were reported in ‘possible TBM’ cases was also calculated.

All analyses were performed in STATA (version 11.2), except for BI, PAI, PABAK and Kmax, which were calculated using WinPepi (Version 11.15) [24].

## Results

**Figure 1:**
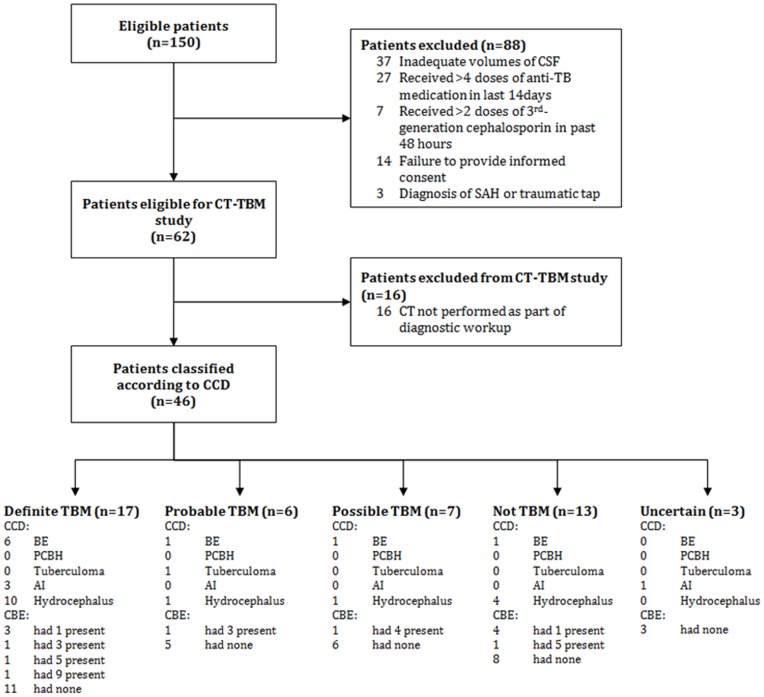
The flow of patients through the study.

Of the 62 patients assessed over the period of this study, 46 met inclusion criteria (See Figure 1). Patient demographics were as follows: 22 male and 24 female; mean age of 33 years (median 31, range 15–57). Human Immunodeficiency Virus (HIV) status was known in 35 (out of 46) patients, and 20 were HIV positive and 15, HIV negative. The status of patients at three months was as follows: alive (30), dead (12) and unknown (4). Of the unknown cases, 2 had been classified as ‘Definite TBM’, 1 as ‘Probable TBM’ and 1 as ‘Unknown’. For the sixteen patients without brain imaging studies excluded from this analysis, the final diagnoses were: Definite TBM (3), Probable TBM (1), Possible TBM (2), Uncertain (2) and Not TBM (6).

### Intra-rater reliability

Complete results for intra-rater reliability are presented in Table 1. The reliability of the criteria for basal meningeal enhancement varied widely, and prevalence of asymmetry was high for all variables. Good agreement was found for ‘contrast filling the cisterns’ ‘double and triple line signs’, “Y’ sign’, ‘join the dots’, ‘infundibular recess of the third’ and ‘asymmetry’, with all of the adjusted values suggesting very good agreement. Most of the criteria for basal meningeal enhancement were significantly affected by prevalence asymmetry, with adjusted kappas (PABAK) showing very good agreement for all but three of the parameters.

**Table 1:** Intra rater Agreement.

Parameters	Kappa	SE	LL95	UL95	Kmax	BI	PAI	PABAK
**Consensus Case Definition**								
Basal Meningeal Enhancement	0.71	0.053	0.61	0.81	1	0	0.15	0.72
Tuberculoma(s)	0.47	0.142	0.19	0.75	0.89	0.01	0.89	0.89
Acute infarction	0.81	0.058	0.69	0.92	0.89	–	–	0.9
Hydrocephalus	0.7	0.046	0.61	0.79	0.91	–	–	0.69
Pre contrast basal hyperdensity	0.5	0.143	0.22	0.78	0.72	0.03	0.9	0.9
**Criteria for BE**								
Contrast filling the cisterns	0.74	0.087	0.57	0.91	0.87	0.02	0.82	0.91
Double and triple line signs	0.7	0.085	0.53	0.87	0.86	0.03	0.78	0.88
Linear enhancement	0.57	0.064	0.44	0.69	0.88	0.05	0.32	0.61
Y’ sign	0.78	0.073	0.64	0.93	1	0	0.77	0.91
Infundibular recess of the third	0.62	0.124	0.37	0.86	0.71	0.03	0.88	0.91
Ill defined edge	0.59	0.069	0.45	0.72	0.91	0.03	0.51	0.7
Join the dots’	0.72	0.118	0.49	0.95	0.94	0.01	0.9	0.95
Nodular enhancement	0.35	0.105	0.15	0.56	0.48	0.09	0.81	0.77
Asymmetry	0.61	0.082	0.45	0.77	0.74	0.07	0.71	0.8
All BE (At least 2)	0.72	0.055	0.61	0.82	0.99	0.01	0.34	0.75

SE = standard error, LL = lower limit, UL = upper limit.

As for the CCD criteria, ‘acute infarction’ showed very good agreement, whilst ‘basal meningeal enhancement’ and ‘hydrocephalus’ had good agreement that did not change significantly with adjustment.

### Inter-rater reliability

In the assessment of inter-rater reliability, the criteria for basal enhancement (see Table 2), ‘contrast filling the cisterns’ ‘double and triple line signs’ ‘Y’ sign’, ‘infundibular recess of the third’ and ‘join the dots’ performed the best, showing moderate agreement. For ‘All BE’ agreement was only fair (0.36, 95%CI 0.19–0.52).

**Table 2:** Inter rater Agreement.

Parameters	Kappa	LL95	UL95
**Consensus Case Definition**			
Basal Meningeal Enhancement	0.32	0.18	0.47
Tuberculoma(s)	0.55	0.17	0.89
Acute infarction	0.51	0.26	0.72
Hydrocephalus	0.63	0.48	0.77
Pre contrast basal hyperdensity	0.21	0.07	0.33
**Criteria for BE**			
Contrast filling the cisterns	0.42	0.2	0.62
Double and triple line signs	0.4	0.15	0.65
Linear enhancement	0.2	0.12	0.31
Y’ sign	0.49	0.24	0.66
Infundibular recess of the third	0.52	0.13	0.74
Ill defined edge	0.24	0.09	0.4
Join the dots’	0.49	0.08	0.87
Nodular enhancement	0.37	0.19	0.52
Asymmetry	0.32	0.13	0.5
All BE (At least 2)	0.36	0.19	0.52

LL = lower limit, UL = upper limit.

The CCD parameters performed only slightly better, with ‘hydrocephalus’ showing good agreement and ‘tuberculoma(s) and ‘acute infarction’ showing moderate agreement. Pair-wise analysis failed to identify a particular rater as the cause of the poor agreement observed.

### Sensitivity and Specificity

In the analysis of ‘definite TBM’ cases compared with cases of ‘not TBM’ (see Table 3), individual criteria for basal enhancement were insensitive (0–29.4%). However, when taken individually, all but ‘linear enhancement’ (61.5%) showed high specificity (92.3–100%).

**Table 3:** Definite TBM (n = 17) vs Not TBM (n = 13).

Parameters	Sensitivity (n)	LL95	UL95	Specificity (n)	LL95	UL95
**Consensus Case Definition**						
Basal Meningeal Enhancement	35.3% (6)	14.2%	61.7%	69.2% (9)	38.6%	90.9%
Tuberculoma(s)	0% (0)	–	–	–	–	–
Acute infarction	17.6% (3)	3.8%	43.4%	100% (13)	75.3%	100.0%
Hydrocephalus	56.7% (10)	37.4%	74.5%	69.2% (9)	38.6%	90.9%
Pre contrast basal hyperdensity	0% (0)	–	–	–	–	–
**Criteria for BE**						
Contrast filling the cisterns	11.8% (2)	1.5%	36.4%	100% (13)	75.3%	100.0%
Double and triple line signs	11.8% (2)	1.5%	36.4%	100% (13)	75.3%	100.0%
Linear enhancement	29.4% (5)	10.3%	56.0%	61.5% (8)	31.6%	86.1%
Y’ sign	17.6% (3)	3.8%	43.4%	92.3% (12)	64.0%	99.8%
Infundibular recess of the third	5.9% (1)	0.1%	28.7%	100% (13)	75.3%	100.0%
Ill defined edge	17.6% (3)	3.8%	43.4%	92.3% (12)	64.0%	99.8%
Join the dots’	5.9% (1)	0.1%	28.7%	100% (13)	75.3%	100.0%
Nodular enhancement	5.9% (1)	0.1%	28.7%	92.3% (12)	64.0%	99.8%
Asymmetry	11.8% (2)	1.5%	36.4%	92.3% (12)	64.0%	99.8%
All BE (At least 1)	35.3% (6)	17.3%	58.7%	61.5% (8)	35.5%	82.3%
All BE (At least 2)	17.6% (3)	3.8%	43.4%	92.3% (12)	64.0%	99.8%

LL = lower limit, UL = upper limit, BE = basal enhancement.

Two of the consensus criteria were not present at all in this cohort (‘tuberculoma(s)’ and ‘pre-contrast basal hyperdensity’). ‘Contrast enhancement’ as defined in the CCD was more sensitive (35.3%) than any of the individual criteria of basal enhancement or ‘all BE’, but less specific (69.2%). ‘Acute infarction’ was insensitive (17.6%) but very specific (100%). ‘Hydrocephalus’ was the most sensitive (56.7%) with only moderate specificity (69.2%).

Analysis of ‘definite TBM’ and ‘probable TBM’ cases compared with those classified as ‘uncertain’ and ‘not TBM’ yielded similar results (see Table 4). Sensitivities of the individual criteria for basal enhancement were low (4.3–26.1%). However, all but ‘linear enhancement’ (68.8%) showed high specificity (93.8–100%).

**Table 4:** Definite and Probable TBM (n = 23) vs Uncertain and Not TBM (n = 16).

Parameters	Sensitivity	LL95	UL95	Specificity	LL95	UL95
**Consensus Case Definition**						
Basal Meningeal Enhancement	30.4%(7)	13.2%	52.9%	75%(12)	47.6%	92.7%
Tuberculoma(s)	4.3%(1)	0.1%	21.9%	100%(16)	79.4%	100.0%
Acute infarction	13%(3)	2.8%	33.6%	93.8%(15)	69.8%	99.8%
Hydrocephalus	47.8%(11)	26.8%	69.4%	75%(3)	47.6%	92.7%
Pre contrast basal hyperdensity	0%(0)	–	–	–	–	–
**Criteria for BE**						
Contrast filling the cisterns	8.7%(2)	1.1%	28.0%	100%(16)	79.4%	100.0%
Double and triple line signs	13%(3)	2.8%	33.6%	100%(16)	79.4%	100.0%
Linear enhancement	26.1%(6)	10.2%	48.4%	68.8%(11)	41.3%	89.0%
Y’ sign	13%(3)	2.8%	33.6%	93.8%(15)	69.8%	99.8%
Infundibular recess of the third	4.3%(1)	0.1%	21.9%	100%(16)	79.4%	100.0%
Ill defined edge	13%(3)	2.8%	33.6%	93.8%(15)	69.8%	99.8%
Join the dots’	4.3%(1)	0.1%	21.9%	100%(16)	79.4%	100.0%
Nodular enhancement	4.3%(1)	0.1%	21.9%	93.8%(15)	69.8%	99.8%
Asymmetry	13%(3)	2.8%	33.6%	93.8%(15)	69.8%	99.8%
All BE (At least 1)	30.4%(7)	15.6%	50.9%	68.8%(11)	44.4%	85.9%
All BE (At least 2)	17.4%(4)	5.0%	38.8%	93.8%(15)	69.8%	99.8%

LL = lower limit, UL = upper limit, BE = basal enhancement.

One of the CCD criteria was not found in this analysis (‘pre-contrast basal hyperdensity’). Sensitivities were very low for ‘tuberculoma(s)’ (4.3%) and ‘acute infarction’ (13%), though specificity was high (93.8–100%). ‘Contrast enhancement’ performed better than any of the individual criteria for basal enhancement (30.4%), with reasonable specificity (75%). ‘Hydrocephalus’ remained the most sensitive (47.8%) with similar specificity (75%).

## Discussion

CT scanning is widely used and forms part of algorithms proposed for distinguishing TBM from other forms of meningitis. Although not a substitute for microbiological diagnosis, CT scanning has the advantages of being non-invasive and quick to perform and report, providing the potential for rapid commencement of treatment and an improved prognosis. However, to date there appears to have been no attempt to examine the diagnostic performance of this method in adults in a systematic and prospective manner. Since radiological diagnoses are descriptive and subject to interpretation it is important to assess both intra- and inter-rater variability since this influences the reliability of the investigation.

The results of our study, based on a sample of 46 adult patients with a diagnosis of meningitis based on clinical and abnormal CSF findings, provides reason for concern about the role of CT scans in the diagnosis of TBM. Our findings suggest that the reliability between radiologists’ interpretation of CT findings taken to be suggestive of TBM is far from optimal. First, although intra-rater agreement is good to very good, meaning that individual reporters provide consistent evaluations of individual scans, inter-rater agreement is poor, which provides the clinician with little confidence in the reported findings, and therefore the strength of diagnosis. Specifically, the fact that basal meningeal enhancement, the finding that is most often associated with TBM, was found to be one of the most unreliable features has important implications. Given the good intra-rater reliability, it may be that experienced readers each had a clear idea of what in their view constituted basal meningeal enhancement, but that these differed one from another.

Furthermore, the results show that of the five major CT features supporting a diagnosis of TBM (hydrocephalus, infarcts, tuberculoma(s), basal meningeal enhancement and the presence of pre-contrast basal hyperdensities), hydrocephalus and meningeal enhancement were the most commonly found consensus features in TBM and have moderate specificity, but that the other features were rare, and if absent, do not rule out TBM.

Finally, we were able to evaluate the significance of the 9 features proposed to support the presence of basal enhancement, which has been emphasized as an important feature of TBM. Intra-rater reliability was good to fair for most of the nine signs, as well as the derived variable (‘all BE’). However, inter-rater reliability was fair (in five features) or poor (in four features) and the derived variable (‘All BE’) among the poorest. Analysis of the sensitivities and specificities revealed low sensitivity (less than 50%) for all but one of the CCD parameters, and all of the BE criteria. Notably, the derived variable, representing the presence of at least two of the signs, showed worse sensitivity than some of the individual signs. Specificity was high for the CCD parameters, and very high for the individual BE criteria.

It is not surprising that the sensitivities of the BE criteria in our study are lower than reported in a study of paediatric patients with TB [12], as basal meningeal enhancement is less often detected in adults than in children with TBM. In a prospective paediatric cohort [25], basal meningeal enhancement was shown to be 82.7% sensitive and 100% specific, whilst the presence of tuberculoma(s) or infarction(s) had low sensitivities (23.6% and 19.3%) but very high specificities (100% and 92.3%). Our results in adults for basal enhancement in TBM are consistent with that of others in which only 8–34% of cases had this feature and 45%, hydrocephalus [4], [11], [12]. However, in contrast to these, one study reports basal enhancement in up to 81.8% of adult, HIV negative patients [15] with TBM. The prevalence of other features in reports of TBM in adults include infarction in up to 44% of patients, and tuberculoma(s) in up to 31% [4], [11], .

The poor inter-rater reliability of basal meningeal enhancement emphasises the need for clearer definitions which may be standardized and validated. In this regard, Przybojewski et al’s attempt at establishing a set of objective criteria, with clear definitions and examples that describe basal enhancement is a step in the right direction [12]. However, our findings suggest that these criteria suffer from similar flaws to the broader descriptions of BE - they have good intra-rater reliability but have poor inter-rater reliability, which reduces confidence in the contribution of these radiological signs to the diagnosis of TBM.

Somewhat concerning is the lack of reliability for both acute infarction - often taken to be of prognostic value - and hydrocephalus, which might signal the need for surgical intervention. Although the numbers are small, all three patients with acute infarction had definite TBM when compared with those who did not have TBM, and this sign may be useful to rule in TBM in the right context.

The strength of the current study lies in its prospective nature. Most studies assessing the value of CT in the diagnosis of TBM have been retrospective. Consequently they have not been able to standardize imaging techniques, the level of training of the imaging reviewers, and the timing of the imaging study in relation to the clinical presentation. Furthermore, the methods for seeking microbiological confirmation of TBM, the gold standard, have varied, and if suboptimal, might have created a bias favouring selection of more severe cases. Similarly, controls were not necessarily selected for the similarity of their clinical presentations – the true clinical dilemma. Instead, in some studies, patients with an established known alternative diagnosis that presented during the same period and had both imaging and CSF analysis, have been used as controls [12]–[14].

There are several limitations to this study: the small sample size does not allow for a comparison of HIV infected versus uninfected patients, and it is possible that the imaging criteria may be different in immunocompromised HIV positive patients. Secondly, patients with TBM and a normal CSF were excluded (though this was probably a small number of cases). Finally, the decision to perform a CT scan in each patient was not according to standard criteria, but at the judgment of the attending physician. Although perhaps not ideal for a clinical study, this represented standard conditions in the study context (a large public referral hospital serving a community with high risk for tuberculosis).

Given that the sensitivity of CT imaging findings in TBM in our series of adult patients was low, and given the lack of reliability of reporting of the features of basal enhancement, the use of separate scoring systems for patients who have had imaging and those that have not must be questioned. Those with imaging are likely to score lower and might be falsely assigned a lower probability of having TBM. On the basis of this study, we recommend that the same scoring system should be used for both categories, that is, regardless of the presence of the CT findings, until the reliability and test performance of CT findings are more widely assessed in a number of relevant clinical settings. In addition, clinicians should be made aware of the need to interpret CT findings in suspected TBM with caution and to rely on the other diagnostic criteria for the presence of TBM. Specifically, it must be emphasized that a normal CT brain scan is not uncommon in early TBM in adult patients, and clinicians should not be falsely reassured by this finding.

Despite the widespread use of CT imaging findings in the diagnosis of tuberculous meningitis, few studies have examined the reliability and diagnostic performance of the most common findings among adults. Based on a prospective cohort of patients with a clinical diagnosis of meningitis, we have shown that the imaging parameters included in a recent consensus case definition, including basal meningeal enhancement, suffer from poor inter-rater reliability and poor to moderate sensitivity and specificity among adult patients. Furthermore, a recent set of criteria which aimed to allow for more objective diagnosis of basal meningeal enhancement was found to be very insensitive in an adult population, and suffered from poor inter-rater reliability. Whilst our numbers are relatively small, these findings suggest that imaging findings should be interpreted with caution, and that further work is needed to derive imaging criteria which are more reliable.

## Supporting Information

Table S1 **Consensus tuberculous meningitis diagnosis.** This table from Marais et al. [9] lists the scoring system for the classification of suspected tuberculous meningitis patients into definite, probable, possible or not TBM.
(DOCX)
