# Supplementary material for: Reliability and Diagnostic Performance of CT Imaging Criteria in the Diagnosis of Tuberculous Meningitis
Source: PLoS One. 2012 Jun 29;7(6):e38982. doi: 10.1371/journal.pone.0038982 (PMC3387202; doi:10.1371/journal.pone.0038982)
Supplement: Table S1 — Consensus tuberculous meningitis diagnosis. This table from Marais et al. [9] lists the scoring system for the classification of suspected tuberculous meningitis patients into definite, probable, possible or not TBM. (DOCX) [file pone.0038982.s001.docx]

**Supporting information:**

| **Table S1. Consensus tuberculous meningitis diagnosis [9]** |
| --- |
| **Clinical entry criteria**   - Symptoms and signs of meningitis including one or more of the following: headache, irritability, vomiting, fever, neck stiffness, convulsions, focal neurological deficits, altered consciousness, or lethargy.   **Tuberculous meningitis classification**  *Definite tuberculous meningitis*   - Patients should fulfill criterion A or B:   A) Clinical entry criteria plus one or more of the following: acid-fast bacilli seen in the CSF; Mycobacterium tuberculosis cultured from the CSF; or a CSF positive commercial nucleic acid amplification test.  B) Acid-fast bacilli seen in the context of histological changes consistent with tuberculosis in the brain or spinal cord with suggestive symptoms or signs and CSF changes, or visible meningitis (on autopsy).  *Probable tuberculous meningitis*   - Clinical entry criteria plus a total diagnostic score of 10 or more points (when cerebral imaging is not available) or 12 or more points (when cerebral imaging is available) plus exclusion of alternative diagnoses. At least 2 points should either come from CSF or cerebral imaging criteria.   *Possible tuberculous meningitis*   - Clinical entry criteria plus a total diagnostic score of 6–9 points (when cerebral imaging is not available) or 6–11 points (when cerebral imaging is available) plus exclusion of alternative diagnoses. Possible tuberculosis cannot be diagnosed or excluded without doing a lumbar puncture or cerebral imaging.   *Not tuberculous meningitis*   - Alternative diagnosis established, without a definitive diagnosis of tuberculous meningitis or other convincing signs of dual disease. |
